# Food Makes the Man

**Published:** 1883-11

**Authors:** 


FOOD MAKES THE MAN.—Speaking roughly says the *Lancet*, about three-fourths, by weight, of the body of man is constituted by the fluid he consumes, and the remaining fourth by the solid material he appropriates. It is therefore no figure of speech to say that food makes the man. We might even put the case in a stronger light and affirm that man is his food. It is strictly and literally true, that "A man that drinks beer thinks beer." We make this concession to the teetotalers, and wili add that good sound beer is by no means a good thought factor, whatever may be the intellectual value of the commodity commonly sold and consumed under that name. It cannot obviously be a matter of indifference what a man eats and drinks. He is, in fact, choosing his animal and moral character when he selects his food. It is impossible for him to change his inherited nature, simply because modifications of development occupy more than an individual life, but he can help to make the particular stock to which he belongs more or less berry, or fleshy, or watery, and so on, by the way he feeds. We know the effect the feeding of animals has on their temper and very natures; how the dog fed on raw meat and chained up so that he cannot work off the superfluous nitrogenized material by exercise becomes a savage beast, while the same creature fed on bread and milk would be tame as a lamb. The same law of results is applicable to man, and every living organisms is propagated "in its kind" with a physical and mental likeness. This is the underlying principle of development. Happily the truth is beginning, though slowly and imperfectly, to find a recognition it has long been denied.

